# Research Advances in MicroRNA-Mediated Regulation of Bamboo Organ Development

**DOI:** 10.3390/plants15111705

**Published:** 2026-05-31

**Authors:** Wenjing Yao, Qin Tan, Hongyue Gu, Rui Zhou, Yulong Ding, Shuyan Lin

**Affiliations:** 1 State Key Laboratory for the Development and Utilization of Forest Food Resources, Nanjing Forestry University, Nanjing 210037, China; yaowenjing@njfu.edu.cn; 2 State Key Laboratory of Tree Genetics and Breeding, Northeast Forestry University, Hexing Road, Harbin 150040, China; 3 Bamboo Research Institute, Nanjing Forestry University, Nanjing 210037, China; tq3172579493@163.com (Q.T.); guhongyue0228@163.com (H.G.); kkismoon@163.com (R.Z.); ylding@vip.163.com (Y.D.); 4 College of Forestry and Grassland, Nanjing Forestry University, Nanjing 210037, China

**Keywords:** bamboo, plant organ development, microRNAs, target genes, miRNA-TFs modules

## Abstract

MicroRNAs (miRNAs) are key regulators of gene expression at the post-transcriptional level, playing multiple roles in plant growth and development, signal transduction, environmental stress response, and secondary metabolite formation. The biological functions of miRNAs are relatively conserved in plants, yet certain miRNAs display regulatory functions and mechanisms that are species-specific. Increasing evidence underscores the significance of miRNA-transcription factor (TF) molecular modules in plant organ development. Compared to other Poaceae plants such as *Oryza sativa*, bamboo (Poaceae: Bambusoideae) exhibits a greater diversity of developmental patterns in organ development throughout its life cycle. However, current research on miRNA-mediated bamboo organ development remains relatively scattered, and the mechanisms of action of key miRNA-TF modules are still poorly understood in bamboo plants. In the review, we outlined the unique biological characteristics of root, shoot, culm, leaf, and flower in bamboo plants and synthesized the research progress on miRNA-mediated regulation of bamboo organ development. Prominently, we focused on the potential regulatory functions of miRNA-TF modules in shaping developmental characteristics of bamboo organs. Last but not least, we summarized the current research limitations in this field and proposed future directions and strategic approaches to facilitate further in-depth exploration. This review not only deepens our understanding of the unique developmental characteristics of bamboo organs but also clarifies the research framework of miRNA-TF modules governing these processes, thereby providing theoretical references for innovative breeding and genetic improvement of bamboo plants.

## 1. Introduction

Bamboo, belonging to the subfamily Bambusoideae of the grass family (Poaceae), is one of the fastest-growing and most versatile plants on earth [1]. It possesses significant economic, ecological, and cultural values, playing a unique role in developing green economy, addressing climate change, and advancing ecological civilization [2]. In recent decades, bamboo has been at the forefront of sustainable design and green technology as a renewable alternative to timbers, bio-composites, biofuel source, and innovative engineering materials [3]. Often mistaken for a tree, bamboo is technically a perennial, woody grass with unique biological characteristics [4]. It is a giant, lignocellulose-abundant grass capable of growing over 90 cm in a single day. Some bamboo species typically reach a height of 30 m but can grow as tall as 42 m under optimal conditions [5]. In addition to its rapid growth, bamboo is also renowned for its exceptional combination of mechanical strength, hardness, lightness, and flexibility. Bamboo culms are woody and hollow between the nodes, yet they exhibit a tensile strength comparable to steel and a strength-to-weight ratio that rivals modern alloys [2,4,5]. Bamboo primarily propagates via its underground rhizome systems. The two general patterns for bamboo growth are sympodial bamboo, which grows in tight clusters, and monopodial bamboo, which can spread aggressively over large areas [2,5]. Bamboo plants have special reproductive habits, characterized by a prolonged vegetative phase, unpredictable flowering time, and rare seed production. After gregarious flowering, most bamboo species decline in growth and even die simultaneously [6]. Owing to these unique biological characteristics, research on the molecular mechanisms of bamboo organ development has lagged significantly behind that of other Poaceae plants, such as *Oryza sativa*. A multitude of questions regarding bamboo organ-specific development have yet to be elucidated, such as rapid growth of bamboo shoots, high lignification of bamboo culms, unpredictability of bamboo flowering, and structural differences between adaxial and abaxial surfaces of bamboo leaves.

MicroRNAs (miRNAs) are a class of non-coding single-stranded small RNA molecules encoded by endogenous genes, typically 18–25 nucleotides in length. The biological functions of plant miRNAs are primarily involved in the essential life processes such as plant organ development, signal transduction, stress responses, and secondary metabolite formation [7]. MiRNAs act as key regulators of gene expression at the post-transcriptional level, negatively regulating target genes through mRNA splicing and translation inhibition in plants. Most target genes of miRNAs are transcription factors (TFs) in plants, which themselves play crucial roles throughout the growth and development process of plants [8]. As primary switches in complex regulatory networks, TFs can activate or repress the transcription of downstream target genes by specifically binding to their cis-elements [9]. Increasing evidence underscores the importance of miRNA-TF molecular modules in plant organ development, such as root apical meristem (RAM) and shoot apical meristem (SAM) formation, flowering and floral organ differentiation, seed germination, seedling development, leaf morphogenesis, root development, and xylem development [8,10]. Although miRNAs tend to have relatively conserved biological functions in plants, certain ones show species-specific regulatory roles and mechanisms [11]. Current studies on miRNA-mediated regulation of bamboo organ development are still in their infancy, and key miRNA-TF modules regulating bamboo growth and development remain enigmatic. In this review, we summarized morphological and developmental characteristics of bamboo organs (e.g., roots, shoots, culms, leaves, and flowers), and outlined current research progress on miRNA-mediated regulation of their development process. Notably, we focused on the potential miRNA-TF modules in shaping unique developmental characteristics of bamboo organs, aim to provide basic theoretical references for forest cultivation, genetic improvement, and molecular breeding of bamboo.

## 2. MiRNA-Mediated Regulation of Bamboo Root Development

### 2.1. Morphological and Developmental Characteristics of Bamboo Root

**Figure 1:**
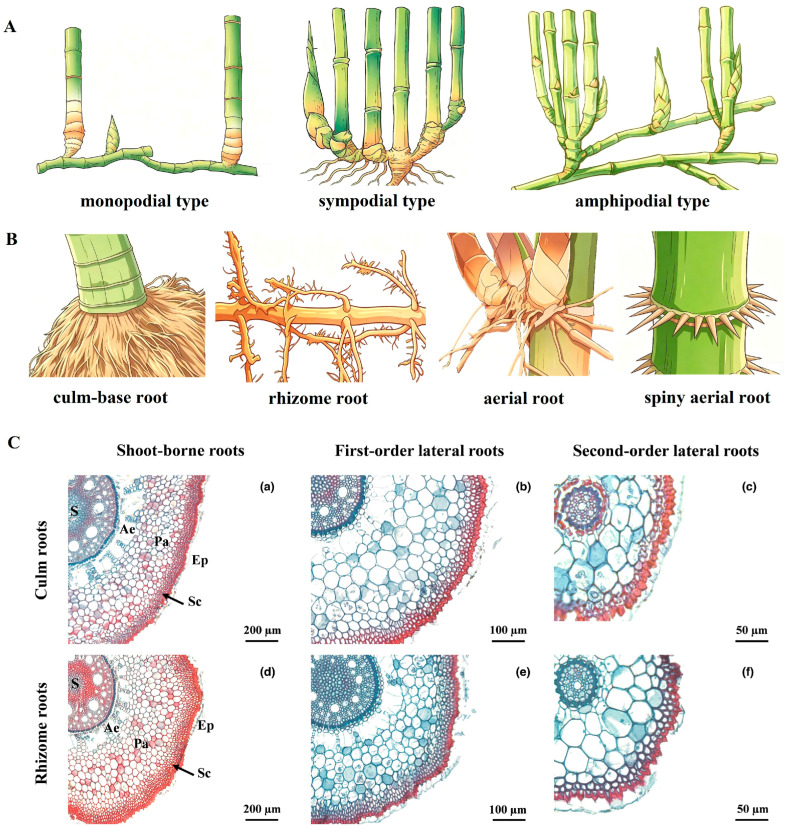
Morphological and developmental characteristics of bamboo roots. (**A**) Three types of bamboo root system. (**B**) Four forms of bamboo root. (**C**) Anatomical structure of culm roots and rhizome roots of *Phyllostachys glauca* [13]. Cross-sections of different root classes of culm roots: shoot-borne root (**a**), first-order lateral root (**b**), second-order lateral root (**c**); cross-sections of different root classes of rhizome roots: shoot-borne root (**d**), first-order lateral root (**e**), second-order lateral root (**f**). S, stele; Ae, aerenchyma; Pa, parenchyma; Sc, sclerenchyma; Ep, epidermis.

Bamboo species can be categorized into three types based on root system, including running bamboo (monopodial rhizomes), clumping bamboo (sympodial rhizomes), and mixed bamboo (amphipodial rhizomes) (Figure 1A). A dense cluster of adventitious roots with similar diameter constitutes the clumping root system, whereas continuously generated rhizome roots form the running root system [5]. The mixed bamboos exhibit both monopodial and sympodial rhizomes [12]. These root systems function in anchoring the plant, absorbing water, minerals, and trace elements from the soil, providing energy, and facilitating vegetative propagation [13]. Bamboo plants generally possess a fibrous root system, characterized by the absence of a primary taproot. The primary roots formed after seed germination undergo rapid physiological senescence, and their absorptive and anchoring functions are substituted by the abundant adventitious roots differentiated from the base of bamboo stools and the nodes of bamboo rhizomes [2]. The surface of adventitious roots is densely covered with root hairs, significantly expanding the root surface area. These adventitious roots further interweave through hierarchical branching, constructing a dense fibrous root network to sustain plant growth and development [14]. Bamboo roots can be classified into four distinct forms according to their developmental origin, including the primary root deriving from the radicle, the culm-base root initiating from the nodes of culm base, the rhizome root emerging from rhizome nodes, and the aerial root originating from culm nodes or branch bases (Figure 1B). Anatomically, the root structure comprises epidermal tissue, parenchyma, and vascular bundles, with a layer of thickened cells between the phloem and pith, a central pith cavity, and cortical aerenchyma. Shoot-borne roots in both culm and rhizome roots differ from first- and second-order lateral roots by having heavily lignified parenchyma cells, which would limit resource acquisition (Figure 1C) [13].

The development of bamboo root system is characterized by distinct spatial stratification and clonal propagation primarily via underground rhizomes [3,12]. Seedling plants of running bamboos typically develop underground rhizomes within six months to a year [2]. The underground rhizomes horizontally grow at a shallow depth of up to 150 mm and produce shoot buds and adventitious roots from the nodes [3]. The thicker the bamboo rhizome, the fewer lateral buds differentiate into rhizome buds. Correspondingly, the greater the number of rhizome nodes, the higher the biomass of underground roots. Most buds on underground stems exist in a dormant state, and lateral bud germination is dominated by rhizome buds [15]. Within a single growing season, the elongation rate of underground rhizomes follows a slow–fast–slow pattern [16]. Moreover, the on-year and off-year phenomenon exerts a significant influence on the growth period of bamboo roots; specifically, the root growth period during the on-year is notably shorter than that during the off-year [16,17]. New root emergence can occur from March to November, yet it is predominantly concentrated in the period from July to October, accounting for 80.18% of annual emergence [17]. The sub-superficial rhizomes and deep penetrating roots form a dense network that efficiently stabilizes the upper forest soil layer [3]. Bamboo root system exhibits a multi-age-class structure, typically comprising three classes, including juvenile roots (less than 1 year old), mature roots (1 to 3 years old), and senescent roots (over 3 years old) [18]. Young rhizome roots are mostly distributed in the 0–20 cm soil layer, whereas aged rhizome roots generally inhabit deeper soil layers ranging from 20 to 60 cm [19].

### 2.2. Research Progress on miRNA-Mediated Regulation of Bamboo Root Development

**Figure 2:**
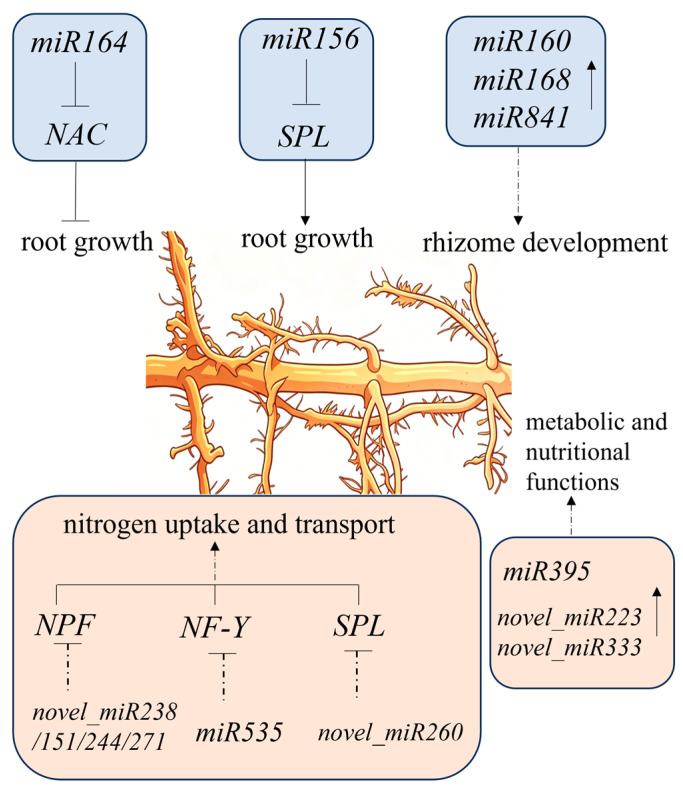
MiRNA-mediated regulation of bamboo root development. Notes: “↑” and “⊥” represent promotion and inhibition, respectively. Solid and dashed lines represent experimental validation and correlation analysis, respectively.

Current understanding of miRNA-mediated regulation in the development of bamboo root and rhizome remains limited, with only a few studies having elucidated. A key example is *ped*-*miR164b* from *Phyllostachys edulis*, which negatively regulates its target gene *PeSNAC1* to modulate root development. The expression of *ped*-*miR164b* displays a decreasing gradient from roots and sheaths to leaves and stems—a pattern opposite to that of *PeSNAC1*. Consistently, Arabidopsis lines overexpressing *ped*-*miR164b* exhibits inhibited root growth, in contrast to the promoted growth in *PeSNAC1*-overexpressing lines [20]. Several auxin-related miRNAs, including *miR160g*, *miR160c*, *miR164d*, and *miR841*, are upregulated in the rhizome of moso bamboo. It suggests that these miRNAs may participate in rhizome development by fine-tuning auxin signaling pathways [21]. Among these regulators, *miR160* has been established as a key mediator of root development by targeting *ARF10*, *ARF16*, and *ARF17* in *Arabidopsis thaliana*. It participates in multiple processes including root cap formation, root gravitropism, lateral root initiation, and primary root elongation. Specifically, the transgenic plants overexpressing *ARF16* exhibit a reduction in lateral root number, whereas miR160c-overexpressing lines show the opposite phenotype [22]. Additionally, several important miRNAs, such as *miRNA*-*156a*, *156b*, and *168*, have also been identified as abundant in the roots of moso bamboo. Particularly, *miR156* shows significant enrichment in the roots of moso bamboo during the vegetative growth phase [21]. Additionally, it is demonstrated that *miR156o*-*SPL3*-*1*/*SPL13*-*2* molecular module promotes root development of moso bamboo [23]. Substantially, the role of *miR156*-SPL module in regulating lateral root development and root nitrogen uptake is also revealed in both apple and Arabidopsis [24]. An investigation comparing bamboo shoot and rhizome shoot revealed 56 upregulated miRNAs in the rhizome shoot of *Phyllostachys praecox*, with members of the *miR395* family being a key group associated with metabolic and nutritional functions [25]. A few miRNAs were also proved to be pivotal regulators in nitrogen metabolism networks in the roots of moso bamboo, participating in the precise control of nitrogen uptake and assimilation by targeting nitrate transporter genes and TFs [26]. For instance, *novel_miR238*, *novel_miR151*, *novel_miR244*, and *novel_miR271* are predicted to regulate nitrogen absorption and transport by modulating the expression of NPF (Nitrate Transporter 1/PepTide Transporter Family) genes. Additionally, *miR535_3p* influences the expression of downstream nitrogen metabolism-related genes by regulating NF-Y TFs. *Novel_miR260* targets SPL TFs, potentially integrating nitrogen metabolism with developmental processes through SPL-mediated signaling. While *novel_miR223* and *novel_miR333* may indirectly regulate the expression of key nitrogen metabolism enzymes or transporters [26]. In summary, the conserved miRNA-TF modules such as *miR164*-NAC and *miR156*-SPL, have been demonstrated to inhibit and promote root development of moso bamboo, respectively. A few miRNA-target genes, such as *miR535*-NF-Y, *novel_miR238*/*151*/*244*/*271*-NPF, and *novel_miR260*-SPL, may play potential roles in root nitrogen uptake in bamboo plants. Several miRNAs, such as *miR160*, *miR168*, *miR841*, *miR395*, *novel_miR223*, and *novel_miR333*, have been identified to be upregulated in bamboo roots, suggesting their potential roles in mediating bamboo root development and nutritional functions (Figure 2).

## 3. MiRNA-Mediated Regulation of Bamboo Shoot Development

### 3.1. Morphological and Developmental Characteristics of Bamboo Shoot

**Figure 3:**
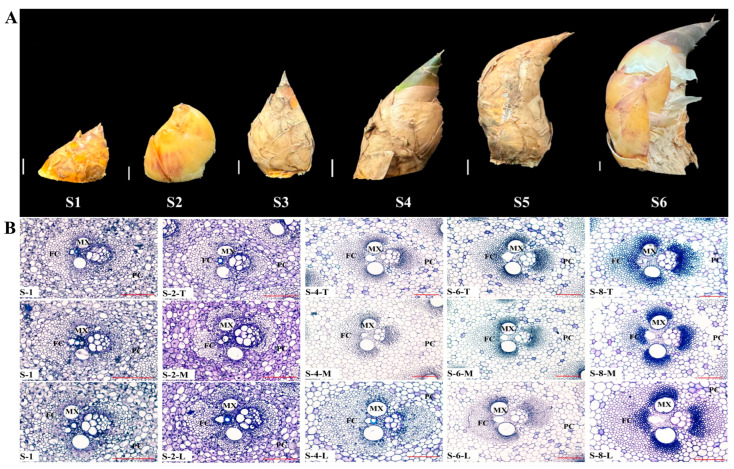
Morphological and developmental characteristics of bamboo shoots. (**A**) Morphological and developmental characteristics of sympodial bamboo shoot [28]. Scale bars = 1 cm. (**B**) Transverse sections of vascular bundle in bamboo shoots with different heights [30]. Notes: T, M, and L, represent the top, middle, and lower portions of the 13th internode, respectively. FC, fiber cells; MX, metaxylem; PC, parenchyma cells. Scale bars = 200 μm.

Bamboo shoot development is a highly dynamic and rapid process characterized by several distinct stages and unique physiological features. It mainly involves the initiation and emergence of shoot buds, followed by the vertical elongation of sprouting shoots [2,14]. The development process of underground bamboo shoot buds can be divided into dormant stage, germination stage, early developmental stage, middle developmental stage, late developmental stage, and mature stage based on morphological characteristics [27,28]. The initiation and emergence of shoot buds refer to the physiological process in which bud primordia on the rhizome break dormancy under suitable conditions and subsequently develop into either germinable shoot buds (Figure 3A, S1) or non-germinable pseudobuds. This process is intermittent, and the number of underground shoot buds gradually increases during the shoot emergence season. The germinable shoot buds enter a developmental period, marked by significant thickening during underground tissue differentiation and maturation (Figure 3A, S2–S5). The shoot buds generally mature and emerge from the soil as shoot apexes protected by multiple layers of tightly wrapped sheaths (abnormal leaves) (Figure 3A, S6) [28]. By the time, the basic structure of aboveground culm has already formed, containing nodes, internode primordia, and undifferentiated intercalary meristem (IcM) [2,14]. Then the sprouting shoots undergo a brief but intense period of vertical elongation growth, which is marked by rapid cell division and elongation, efficient resource mobilization, and a tightly regulated transition from a soft, edible shoot to a rigid, woody culm. The phase is driven primarily by IcM activity at the base of each internode, which is differentiated from the apical meristem of rhizome lateral buds [27,28].

The activity of the apical meristem is also crucial for the thickening growth of bamboo shoots. Since bamboo lacks a vascular cambium and does not undergo secondary growth, the ultimate diameter of the culm is determined solely during the primary thickening phase of the shoot [2,14]. Thickening growth primarily results from an increase in both the number and volume of pith tissue cells, which derive from the central region of SAM [29]. Concurrently, the shoots undergo tissue differentiation, during which soft parenchymatous tissues gradually develop into the hardened lignified culm walls [29,30]. The lignification process of bamboo shoot can be divided into three stages: Stage I (height of 1.0 m to 2.0 m): The lignification rate is extremely slow and most cells are in the division and elongation stages. Stage II (height of 2.0 m to 6.0 m): The lignification rate accelerates and cell wall thickening becomes dominant. Stage III (height exceeding 6.0 m): The lignification process proceeds continuously, cell elongation ceases completely, and the shoots gradually develop into the culms with high lignin content (Figure 3B) [30]. Bamboo shoot development is accompanied by various physiological and biochemical changes, such as carbohydrate metabolism and energy conversion, which are supported by the efficient remobilization of stored nutrients from the rhizome and culm system [27,31].

### 3.2. Research Progress on miRNA-Medicated Regulation of Bamboo Shoot Development

**Figure 4:**
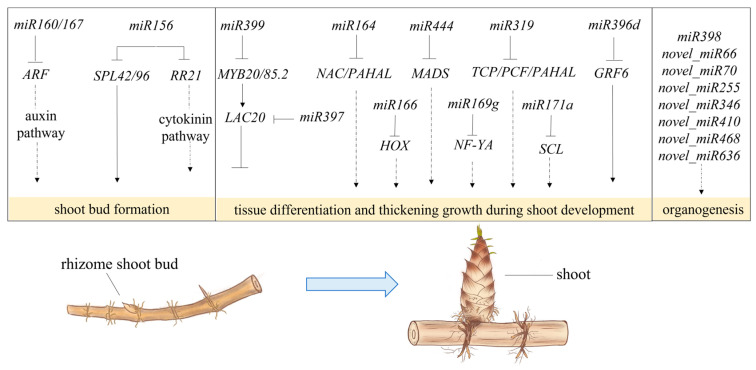
MiRNA-mediated regulation of bamboo shoot development. Notes: “↑” and “⊥”represent promotion and inhibition, respectively. Solid and dashed lines represent experimental validation and correlation analysis, respectively.

Bamboo shoot development is tightly controlled by a complex interplay of various genetic factors, hormonal signals, and environmental cues, which ensure its continuity and accuracy [3,12]. Emerging evidence indicates that miRNAs act as master regulators in the process, orchestrating phytohormone metabolic balance, bud tissue differentiation, and the uptake, transport, and allocation of nutrients. Numerous miRNAs associated with meristematic activity and morphogenesis have been identified to participate in the biosynthesis and signaling pathways of key endogenous hormones in bamboo shoots [20,25,32,33,34]. In the comparative study between bamboo shoot and rhizome shoot, a total of 64 bamboo shoot upregulated miRNAs were revealed in *Phyllostachys praecox*. Meristem and morphological development related miRNAs were most important in bamboo shoot, especially *miR171* and *miR156* members. Additionally, *miR395* members were identified to be primarily linked to metabolism and nutrition, supporting nutrient-dependent shoot growth [25]. Known miRNA families such as *miR156*, *miR164*, *miR319*, and *miR398*, as well as novel miRNAs such as *novel_66*, *novel_70*, *novel_255*, *novel_346*, *novel_410-*, *novel_468*, and *novel_636*, have been identified to participate in shoot organogenesis of *Dendrocalamus latiflorus*. Notably, it was demonstrated that the *miR156*-*SPL42*/*96* module positively participates in shoot organogenesis, and the transgenic bamboos overexpressing *miR156* promote the initiation of bamboo bud organs. *RR21*, a target gene of *miR156*, plays a regulatory role in the cytokinin (CTK) signaling pathway [32]. Additionally, *miR164* plays a negative role in SAM maintenance and gibberellin (GA) biosynthesis, and its target NAC genes are mainly involved in the formation and maintenance of apical meristems and the establishment of organ boundaries [20]. Specifically, a regulatory network of miRNA-mediated “TF-enzyme genes” for shoot lignification in moso bamboo indicated *Ped*-*miR399j*-*5p* and *Ped*-*miR397* suppress the expression of *PeMYB20*/*85.2* and the laccase gene *PeLAC20* in young internodes, respectively. As these miRNAs decline, PeMYB20/85.2 proteins activate *PeLAC20* expression to drive lignification [30]. As many as 68 differentially expressed miRNAs were identified in the underground thickening shoot between moso bamboo and its thick wall variant. Several miRNA-mRNA modules, such as *ped*-*miR171a*-*PeSCL27*, *ped*-*miR319b*-*PePCF*/*PePAHAL*, *ped*-*miR166a*-*PeHOX32*, *ped*-*miR444c*-*PeMADS27*, *ped*-*miR169g*-*PeNF*-*YA4*, *ped*-*miR396d*-*PeGRF6*, and *ped*-*miR164b*-*PePAHAL2* were identified to participate in tissue differentiation and thickening growth of underground buds. Notably, *ped*-*miR160* and *ped*-*miR167* participates in the auxin (IAA) signaling pathway by repressing *PeARF18* and *PeARF17*, respectively [33]. Notably, it has been demonstrated that *ped*-*miR396d* negatively regulates growth-regulating factor (GRF) genes, *PeGRF6*, participating in the primary thickening process of bamboo shoots through multiple regulatory pathways, thereby optimizing tissue development and differentiation in moso bamboo [34]. In conclusion, *miR156*-*SPL*, *miR399*-*MYB*-*LAC*, and *miR396*-*GRF* modules have been confirmed to function in bamboo shoot development. Several miRNA-mRNA pairs, such as *miR164*-*NAC*, *miR160*/*167*-*ARF*, and other miRNA-TF modules are key candidates in regulating bamboo shoot development. It is hypothesized that several known miRNAs and a number of novel miRNAs participate in shoot organogenesis (Figure 4).

## 4. MiRNA-Mediated Regulation of Bamboo Culm Development

### 4.1. Morphological and Developmental Characteristics of Bamboo Culm

**Figure 5:**
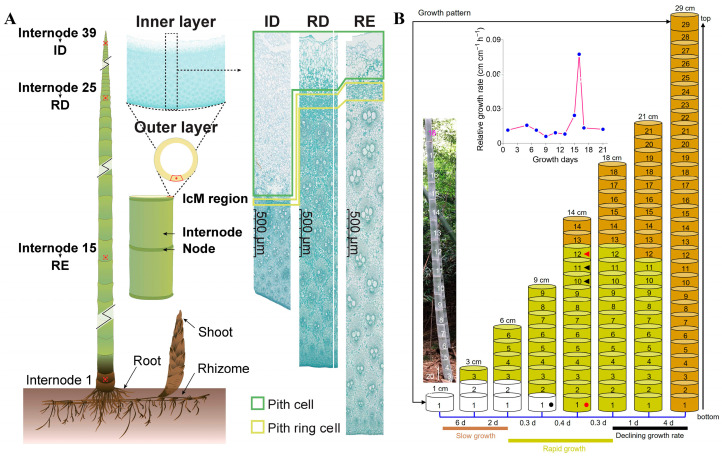
Morphological and developmental characteristics of bamboo culms. (**A**) Three critical developmental stages of intercalary meristems activity: initial cell division phase (ID), rapid cell division phase (RD), and rapid cell elongation phase (RE) [35]. (**B**) Spatiotemporal dynamics of internode growth, represented as stacked cylinders illustrating developmental zones. Zones include division zone (DZ, white), elongation zone (EZ, yellow−green), and maturation zone (MZ, gold). A black spot indicates retrograde cell division, a red spot indicates the end of cell division, black triangles indicate retrograde cell elongation, and a red triangle indicates the end of cell elongation [37].

Bamboo culm development mainly involves internode elongation, lignification, and aging, and exhibits unique developmental patterns and distinct morphological characteristics [2,14]. Based on the activity of IcM, the development of bamboo culm is widely categorized into three distinct phases: initial cell division (ID) phase, rapid cell division (RD) phase, and rapid cell elongation (RE) phase (Figure 5A) [35]. Rapid internode elongation of bamboo culm is a concentrated, single-event process, which occurs almost exclusively during a brief, vigorous elongation phase immediately following shoot emergence. It is the most prominent biological characteristic of bamboo culm development, resulting in significant increases in both height and diameter. For instance, some bamboo species can exhibit a daily growth potential exceeding 114 cm, reach heights of 15–30 m in merely 2–4 months, and achieve full maturity within 3–8 years (Figure 5B) [35,36,37]. Rapid vertical growth of bamboo culm is achieved through synchronized cell division and elongation within each internode, over which SAM exerts critical control [36,37,38,39]. A reduced SAM leads to decreased levels of key hormones like CTK and IAA, downregulating associated signaling pathways and functional gene expression, ultimately resulting in a dwarfed culm with reduced diameter and biomass [36,37]. During this process, a dynamic hormonal interplay sustains cell division, promotes expansion, and enables cell wall remodeling [12]. Phytohormones such as GA, CTK and IAA accumulate in the shoot apex to sustain meristematic activity, whereas stress-related hormones like abscisic acid (ABA), salicylic acid (SA), and jasmonic acid (JA) are more abundant in the lower culm, promoting cell wall thickening and defense gene expression [2,3]. Once primary growth ceases and culm undergoes maturation and lignification, no further vertical or radial secondary growth occurs due to the absence of a true vascular cambium [38,39]. In contrast, cell wall thickening in bamboo culms is a continuous process that spans the entire growth period and persists even after culm reaches maturity [2,14]. Following the completion of height growth, bamboo enters a multi-year maturation phase, during which key culm characteristics including height, volume, and wall thickness remain stable and undergo no significant further change [2,14]. Upon reaching a certain age, bamboo culms undergo senescence and eventual death. A prevailing hypothesis attributes the aging process to the progressive blockage and eventual collapse of the internal transport system within the culm [2,14].

The high degree of culm lignification also represents one of the most distinctive growth traits in bamboo plants, which is the core biological process underlying the formation of wood properties in mature bamboo [40]. The gradient in vascular bundle distribution (denser on the outside) and differential lignification creates a lightweight yet mechanically efficient cylinder, ideal for a fast-growing grass that reaches tree-like heights (Figure 5A) [2,4,5,35]. The lignification degree and lignin content, along with other properties such as fiber length, cell diameter, and bulk density, change with bamboo culm height. Lignin deposition in bamboo culms exhibits a distinct bidirectional gradient. The older bottom internodes contain higher lignin levels than the upper ones (Figure 5B) [37]. Conversely, lignin content decreases from the outer to the inner wall within an individual internode. After bamboo culms reach their maximum height, most parenchyma cells initiate lignification process [40,41]. The properties, such as lignin content and the productivity of entire clump, change as bamboo ages from shoot to mature culm over several years [2,14]. Essentially, the development of culm lignification involves the rapid thickening of secondary cell walls and the ordered deposition of structural components including lignin, cellulose, and hemicellulose, which transforms the culms from a tender state into a rigid and tough structural material [39,40,41].

### 4.2. Research Progress on miRNA-Mediated Regulation of Bamboo Culm Development

**Figure 6:**
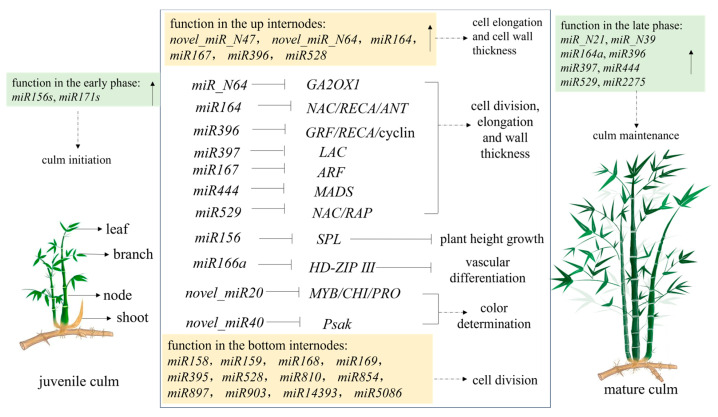
MiRNA-mediated regulation of bamboo culm development. Notes: “↑” and “⊥” represent promotion and inhibition, respectively. Solid and dashed lines represent experimental validation and correlation analysis, respectively.

Bamboo culm development requires the coordination of complex gene regulatory networks, hormone signaling pathways, and environmental factors, as well as energy supply and nutrient allocation [2,3,39]. The research by Jin et al. (2016) highlighted the critical role of novel gene interactions and whole-genome duplication in driving morphological innovation, proposing these mechanisms as molecular foundation for the evolution of rapid-growth traits in woody bamboo [25]. Approximately 55~64 types of miRNAs participate in the critical biological processes such as phytohormone signaling, cell cycle progression, cell wall metabolism, and cell morphogenesis in the initiation and maintenance of bamboo culm growth [25,42,43]. For example, *miR156* and *miR171* family members show high expression during the very early phase, indicating their crucial roles in initiating the development of aboveground bamboo culm [25]. In particular, *phe*-*miR156o* represses the expression of *PheSPL3*-*1* and *PheSPL13*-*2* to regulate culm growth in moso bamboo. Overexpression of *phe*-*miR156o* could remarkably delay the plant height growth of transgenic rice [23]. Conversely, several miRNAs such as *miR158*, *miR159*, *miR168*, *miR169*, *miR395*, *miR528*, *miR810*, *miR854*, *miR897*, *miR903*, *miR1439* and *miR5086* are upregulated in the late development phase, which likely support the maintenance of bamboo culm growth [3,42]. Similarly, distinct miRNA expression profiles govern internode elongation process in different regions. For instance, *novel_miR_N47*, *novel_miR_N64*, *miR164*, *miR167*, *miR396*, and *miR528* are expressed at lower levels primarily in the bottom internodes, where they regulate cell division. Meanwhile, *novel_miR_N21*, *novel_miR_N39*, *miR164a*, *miR396a*, *miR397*, *miR444*, *miR529*, and *miR2275* facilitate cell elongation and cell wall thickness in the upper internode [43]. Particularly, *novel_miR_N64* targets *GA2OX1*, *miR164* suppresses *RECA*, *ANT*, and *NAC* genes, *miR396* represses *GRF*, *RECA*, cyclin, and xyloglucan-related genes, and the expression pattern of *miR397* is opposite to its target laccase gene [43]. Comparative studies of wild-type moso bamboo and its thick-walled variant reveal that the differential expression of *miR166* family members underlies their distinct vascular bundle patterns. *miR166a*-*3p*, enriched in xylem tissues, regulates HD-ZIP homologs and protein kinases, forming a complex network that integrates developmental and hormonal signals to affect vascular differentiation in moso bamboo [44]. Its role is evolutionarily conserved, as shown in Arabidopsis where *miR166* overexpression downregulates *ATHB15*, *PHB*, *PHV*, and *ATHB8*, causing xylem expansion [11]. Additionally, recent research has significantly advanced our understanding of how miRNAs regulate bamboo culm color. During the greening process of *Phyllostachys vivax*, *novel_miR20*, and *novel_miR40* are downregulated, leading to the upregulation of their target genes, including *MYB_c58701.graph_c1*, *CHI*, *PRO*, *Psb27*, and *PsaK*. Conversely, *novel_miR31*, *novel_miR67*, and *novel_miR75* are upregulated, accompanied by the increased expression of their corresponding targets [45]. In conclusion, multiple conserved and novel miRNAs have been identified to regulate bamboo culm development in different growth phase across different regions. Notably, *miR156*-*SPL* has been proved to inhibit bamboo culm growth, *miR166a*-*HD*-*ZIP* III has been confirmed to promote vascular differentiation in bamboo culm. It is inferred *miR_N64*-*GA2OX1*, *miR164*-*RECA*/*ANT/NAC*, *miR396*-*GRF/RECA/cyclin*, and *miR397*-*LAC* modules likely regulate internode elongation, *novel_miR20*-*MYB/CHI/PRO* and *novel_miR40*-*PsaK* are probably involved in culm color determination (Figure 6).

## 5. MiRNA-Mediated Regulation of Bamboo Leaf Development

### 5.1. Morphological and Developmental Characteristics of Bamboo Leaf

**Figure 7:**
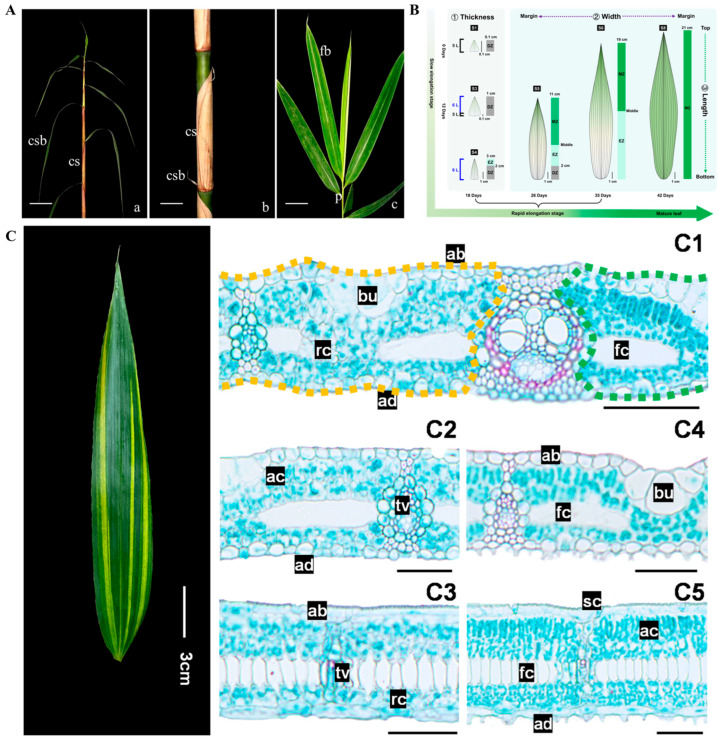
Morphological and developmental characteristics of bamboo leaves. (**A**) Culm sheath and foliage leaf of *Fargesia yunnanensis* [46]. (**a**) Culm sheath at the top of bamboo culms. Scale bar =  10 cm; (**b**) Culm sheath at the middle of bamboo culms. Scale bar  =  10 cm; (**c**) Foliage leaf blade. Scale bar  =  1 cm. Notes: cs, culm sheath; csb, culm sheath blade; fb, foliage leaf blade; p, petiole. (**B**) Anatomical growth model of *Sasaella kogasensis* ‘Aureostriatus’ leaves [49]. Notes: DZ, division zone; EZ, elongation zone; MZ, mature zone; L, cell layer count. (**C**) Anatomical structures of leaf blade in *S. kogasensis* ‘Aureostriatus’ [50]. Notes: Transverse section of leaf blade (**C1**), transverse (**C2**) and longitudinal (**C3**) sections of yellow-zone, and transverse (**C4**) and longitudinal (**C5**) sections of green-zone. Scale bars = 50 μm. Notes: ab, abaxial epidermis; ac, arm cells; ad, adaxial epidermis; fc, fusoid cells; rc, rosette cells; sc, short cells; tv, tertiary vein.

Bamboo leaf organs are distinguished as vegetative (branches and leaves) and culm leaves (culm sheath) (Figure 7A) [46]. Most bamboo species continuously produce new leaves while shedding old ones, with a yearly period of concentrated leaf fall. Leaf lifespan and replacement timing vary by species and climate. A complete bamboo leaf consists of leaf sheath, ligule, auricle, pseudopetiole, and leaf blade (Figure 7A) [46,47]. Bamboo leaves develop from the leaf primordium, first forming leaf sheaths that wrap around the young branches. Then, leaf blade and pseudopetiole emerge from the tip of leaf sheath. Leaf development proceeds in an acropetal direction, meaning that young leaves remain tightly rolled along the midrib as they extend upward [48]. Bamboo leaf development can be divided into three growth sections, primarily categorized into the division zone (DZ), the elongation zone (EZ), and the maturation zone (MZ) [49]. The DZ is located at the leaf base, where cells undergo rapid division. The EZ is adjacent to the DZ, where cells rapidly expand. Above the DZ lies the MZ, characterized by the completion of cellular elongation (Figure 7B) [49]. Increasing numbers of studies have provided detailed insights into the anatomical structure of bamboo leaves, which correlates with their physiological function and environmental adaptation. The anatomical structure of bamboo leaves comprises of epidermis, ground tissue, and vascular bundles (Figure 7C) [50]. The epidermis is composed of a single layer of cells across different bamboo species, and the bulliform cells on the adaxial epidermis serve as a taxonomically significant characteristic [51]. The ground tissue mainly includes two kinds of cells: mesophyll cells and fusiform cells. Differentiated mesophyll cells exhibit a finger-like shape adjacent to the adaxial epidermal layer and a plum-like morphology near the abaxial epidermal layer. Fusiform cells are colorless, transparent short cells located on both sides of vascular bundles, with a nearly rectangular shape (Figure 7C) [50]. Their distribution and morphology represent as key diagnostic features for identifying bamboo species [48,49,50,51]. Recent research indicates moso bamboo features a larger leaf area, bulliform cell area, and vascular bundle area, along with thicker leaves, mesophyll and lower epidermis. The structural advantages enhance photosynthetic efficiency, thereby accumulating more assimilates for culm growth [52]. Additionally, several histological structures of moso bamboo, such as fewer fusiform cells, small bulliform cells, low stomata density, and more trichomes, may improve leaf cold tolerance [53].

Culm sheath is a modified branch that directly connects to culm node via vascular bundles, playing crucial roles in supporting and protecting bamboo shoots (Figure 7A) [46]. The structural characteristics of culm sheath vary across ecological regions and are also considered as valuable trait for bamboo taxonomic identification [3,12,46]. Anatomically, the epidermis of culm sheath lacks bulliform cells or trichomes, and its elongated cell shapes closely resemble those found in branches. Additionally, the vascular bundles of culm sheath also exhibit great similarity in shape and anatomical structure to those of branches [46]. Culm sheath is characterized by its large surface area, possessing the capabilities of photosynthesis and water/nutrients transportation. Its base is primarily the main site for respiration and metabolism, contributing to starch storage, hormone signaling, and sugar catabolism throughout the growth and development of bamboo shoots [2,14,54]. Once bamboo shoot growth nears completion, the culm sheaths rapidly age and die, likely triggered by sugar accumulation within the sheath [55].

### 5.2. Research Progress on miRNA-Mediated Regulation of Bamboo Leaf Development

**Figure 8:**
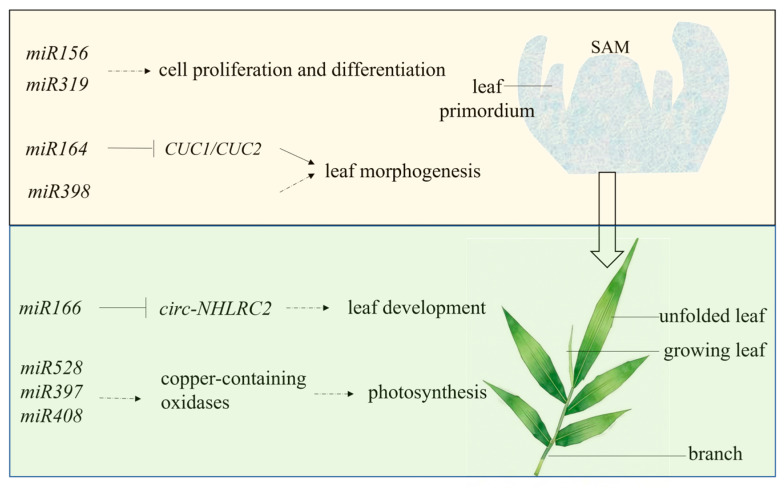
MiRNA-mediated regulation of bamboo leaf development. Notes: “↑” and “⊥” represent promotion and inhibition, respectively. Solid and dashed lines represent experimental validation and correlation analysis, respectively.

Bamboo leaf development requires the coordinated regulation of numerous genes, hormones and environmental factors. Although research on miRNA-medicated regulation of bamboo leaf development remains sparse, several key miRNAs have been identified to play critical roles in bamboo leaf development, orchestrating processes from primordial initiation to photosynthetic maturation. For example, a total of 92 known and 95 novel miRNAs were identified from the leaves of moso bamboo. Among them, *miR397*, *miR1432*, and *miR7748* were identified to be specific conserved in the leaf of moso bamboo [56]. *miR156* and *miR319* were identified to regulate cell proliferation and differentiation timing in leaf formation, while *miR164b*, *miR319b*, and *miR398* were identified to participate in leaf morphogenesis in ma bamboo [32]. Notably, *miR164b* from moso bamboo has been confirmed to modify leaf morphogenesis by regulating the expression of *CUC1* and *CUC2* [57]. Most miRNAs primarily indirectly influence leaf development by regulating the target genes related to photosynthesis and other growth processes. For instance, leaf-preferential miRNAs in moso bamboo, such as *miR528*, *miR397*, and *miR408*, target different copper-containing oxidases (e.g., laccases, plastocyanin-like proteins, polyphenol oxidases), which may fine-tune stress responses and copper allocation to optimize photosynthesis [21]. A small RNA library from the leaf tissues of *D. latiflorus* identified 84 known and 81 novel miRNAs, with 162 potential target genes predicted for the novel group. Key candidates among them target ARF TF and photosynthesis-related genes, linking miRNA activity to hormonal signaling and chloroplast function [58]. Additionally, *miR166* modulates leaf development by targeting and cleaving *circ*-*NHLRC2*, which in turn regulates the genes related to miRNA biogenesis or function. The miRNA-circRNA-mediated module participates in gene expression and metabolic networks of hormonal signaling, environmental stress and homeostasis during leaf growth and development [59]. Summing up the related researches, several miRNAs and only two miRNA-mRNA modules including *miR164b*-*CUC1*/*2* and *miR166*-*circ*-*NHLRC2*, have been identified to regulate bamboo leaf development (Figure 8).

## 6. MiRNA-Mediated Regulation of Bamboo Flowering and Floral Organ Development

### 6.1. Morphological and Developmental Characteristics of Bamboo Flowering and Floral Organs

Bamboo is a perennial one-time flowering plant with unique flowering habits. It exhibits prolong vegetative growth and unpredictable flowering time, which distinguishes it from annual herbs and perennial woody plants [60]. Flowering cycles vary widely among bamboo species, ranging from annual blooming to intervals as long as 120 years. Notably, variation also occurs among populations within the same species [61]. Bamboo flowering generally follows three patterns, including gregarious flowering, partial flowering and sporadic flowering [62]. In the case of gregarious or partial flowering, nearly all the individuals or a large proportion across a wide geographical area or genetic clone synchronously bloom within a period of one to several years. The flowering patterns lead to a series of abnormal morphological and physiological changes. For example, the germination rate of new underground rhizomes and the sprouting rate of aboveground shoots decrease markedly; the chlorophyll content in leaves declines sharply or even leads to leaf abscission; the levels of nitrogen-containing compounds and the carbon-to-nitrogen ratio in rhizomes descend significantly; and the root systems gradually shrink and necrotize. Subsequently, the majority of flowering culms tend to die within one to two years, ultimately triggering mass mortality and even the decline of bamboo forests [60,61,62,63]. By contrast, sporadic flowering is characterized by scattered and random flowering culms within a bamboo population over a longer span of one to several decades [6,62]. After flowering, bamboo forests typically undergo rejuvenation and regeneration through two modes: sexual reproduction and asexual reproduction [6]. Several bamboo species, such as moso bamboo, can produce large quantities of seeds after blooming. Seeds originating from different flowering individuals develop into seedlings of different ages, forming bamboo forests with varying clones [64]. In contrast, many species that rarely produce seeds (e.g., *Bambusa emeiensis*) or have no seeds (e.g., *Shibataea chinensis*) mainly renew bamboo forest through asexual rejuvenation slowly. In these species, buds on the rhizomes of flowering bamboo sprout to form dwarf, weak culms that typically bloom in the same year and generate new underground rhizomes. The buds on these new rhizomes produce further dwarf, leafy-flowering culms in subsequent years, eventually forming a normal, non-flowering forest after several years [6,64].

**Figure 9:**
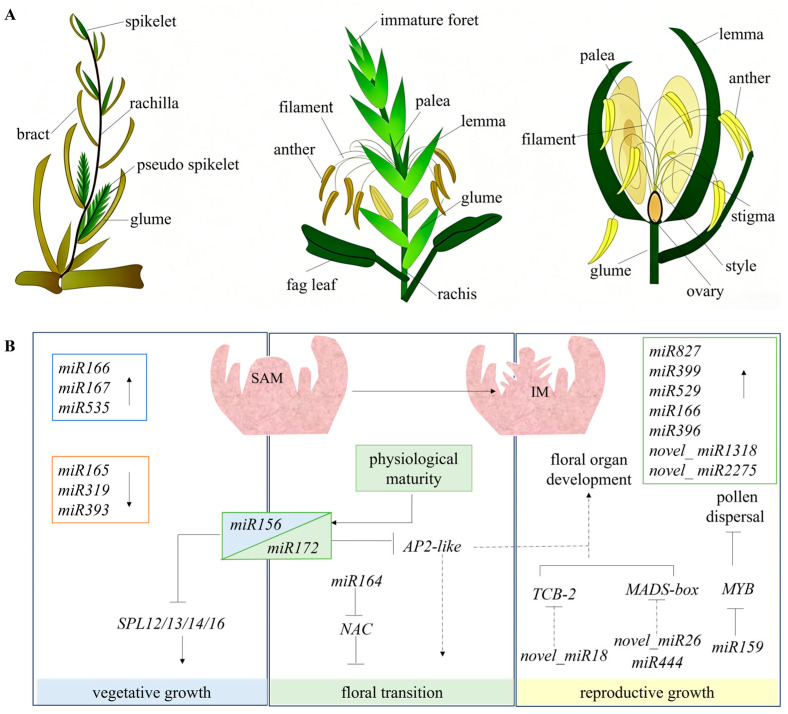
Morphological structure of bamboo floral organs and miRNA-mediated regulation of bamboo flowering and floral organ development. (**A**) Morphological and anatomical structure of bamboo floral organs. (**B**) MiRNA-mediated regulation of bamboo flowering and floral organ development. Notes: “↑” and “⊥” represent promotion and inhibition, respectively. Solid and dashed lines represent experimental validation and correlation analysis, respectively.

Enormous progress has been achieved in understanding the morphological and anatomical structure of floral organs in different bamboo species. Bamboo inflorescences can be classified as indeterminate or determinate, with the spikelet constituting their fundamental unit. Inflorescences vary widely in external morphology and flowering sequence within a spikelet differs significantly among different bamboo species, which are closely related to their special biological characteristics and play a critical role in bamboo taxonomy [65,66]. The structure of bamboo spikelet is generally consistent with that of other Poaceae plants, consisting of lemma, palea, stamens, pistils, and lodicules [2,12]. The lemma is characterized by multiple veins, while the palea bears two keels on its abaxial surface. There are commonly three or six stamens, with slender filaments and bilocular anthers. The pistil is solitary, consisting of an ovary and a single style, topped by two or three feathery stigmas (Figure 9A) [65,66]. The developmental processes of flower buds in the early stage are consistent across bamboo plants, initiating in a tightly coordinated and sequential order: lemma, palea, lodicule, stamens, and pistil [65,66,67]. The widespread phenomenon of gametophyte sterility is one of the unique reproductive characteristics in bamboo plants. Pollen grains of some bamboo species are often prone to sterility and tend to lose viability easily. In addition, the stigmas of a few bamboo species exhibit non-receptivity [64]. These are core intrinsic factors resulting in a remarkably low pollination rate, which consequently leads to the generally low natural seed-setting rate observed in many bamboo species.

### 6.2. Research Progress on miRNA-Mediated Regulation of Bamboo Flowering and Floral Organ Development

Bamboo flowering process is orchestrated by an evolutionarily conserved genetic program that can be modulated by environmental stresses and hormonal balances. The currently accepted model explains bamboo flowering through an intrinsic genetic timer, that brings it to a physiological state of “readiness” to flower. Environmental stresses or resource limitations act as triggers to initiate flowering, sometimes prematurely [60,61,62]. Increasing evidence has demonstrated that the regulatory network constituted by miRNAs and TF target genes exerts crucial stage-specific regulatory functions in floral organ development and vegetative-to-reproductive transition in bamboo plants. Among them, MYB and MADS-box family genes play a predominant role in directing floral organ development [68,69,70,71,72]. Sixteen conserved miRNAs were identified to be specifically and highly expressed in stamens of moso bamboo, including *miRNA159* and *miRNA166*. *Phe*-*miR159* shows high expression in the anthers of moso bamboo, whereas its target genes, *PheMYB98* and *PheMYB42*, exhibit relatively low expression levels. Heterologous overexpression of *Phe*-*miR159* in *A. thaliana* led to indehiscent anthers, inhibited pollen shedding, and consequently reduced fertility [68]. In *D. latiflorus*, a total of 118 conserved and 47 novel miRNAs were identified in developing flowers. Five miRNAs, including *dla*-*miR396e*, *dla*-*miR1318*, *dla*-*miR2275a*, *dla*-*miR2275b*, and *dla*-*miR18*, exhibit distinct expression changes during flowering process, with the novel miRNA, *dla*-*miR18*, showing particularly marked differences in transcript abundance. *Dla*-*miR18* targets *TBC*-*2* gene, which encodes a conserved Rab GTPase-activating protein and participates in programmed cell death, playing a major role in the late-stage development of inflorescences in ma bamboo. Additionally, *dla*-*miR172b* was identified to target *unigene36290_All*, which was annotated as a AP2-like TF. And *dla*-*miR26* and *dla*-*miR444* were identified to regulate the formation and development of floral organs (e.g., sepals, petals, stamens, and carpels) by targeting MADS-box genes [69]. Likewise, as many as 64 known miRNAs and 492 novel miRNAs were identified to be differentially expressed between non-flowering and flowering leaves of moso bamboo. Several conserved miRNAs, including *miR164a*, *miR166a*, *miR167a*, and *miR535a*, exhibit high expression levels in non-flowering plants of moso bamboo, in contrast to the notably low levels of *miR165a*-*3p*, *miR319b*, and *miR393b*-*3p* [70]. Prominently, the expression of *Phe*-*miR164a* is negatively correlated with that of its target NAC genes. Furthermore, the transgenic lines overexpressing *Phe*-*miR164a* exhibit a delayed flowering phenotype. Accordingly, *miR164a* modulates the spatiotemporal expression of NACs, thereby regulating inflorescence development and the establishment of organ primordium boundaries [71]. The study on *Pleioblastus pygmaeus* has identified 11 significantly differentially expressed miRNAs between vegetative and floral buds, targeting 124 TFs. Notably, *miR156a* mediates the repression of SPL members (e.g., *PpSPL12/13/14/16*), thereby delaying vegetative growth, which reveals the core role of age-dependent genetic pathway in floral transition of bamboo plants [72]. Summed up, a variety of conserved miRNAs and novel miRNAs have been identified to regulate floral organ development and floral transition in bamboo plants. The modules such as *novel_miR18*-*TBC*, *miR159*-*MYB*, and *miR26*/*444*-*MADS* likely contribute crucially to bamboo floral development, while *miR156*-*SPL*, *miR172*-*AP2*, and *miR164*-*NAC* may function as essential cascades in controlling bamboo flowering. Notably, the biological function of three modules, including *miR159*-*MYB*, *miR156*-*SPL*, and *miR164*-*NAC*, have been experimentally validated in bamboo plants (Figure 9B).

## 7. Conclusions and Future Prospects

A growing body of research has established miRNAs as critical regulators in plant growth and development. Through the precise modulation of key target genes, miRNAs influence a diverse array of life processes, spanning from root formation, stem elongation, and leaf morphogenesis to flowering timing, fruit development, and seed germination. Summing up the latest findings, relevant research has gradually accelerated progress in identifying miRNA families, predicting their target genes, and elucidating their biological functions in bamboo organ development. Nevertheless, the scope of investigation remains notably constrained. Current research mainly focuses on several economically important bamboo species such as *P. edulis* and *D. latiflorus*, with a lack of attention to unique and rare bamboo species. Furthermore, the primary research emphasis lies in identifying conserved and novel miRNAs and predicting their target genes. In contrast to well-established model plants (e.g., *O. sativa* and *A. thaliana*), which have extensively characterized regulatory networks across all developmental stages, the research on biological functions of bamboo miRNAs is still limited to heterologous expression studies of conserved bamboo miRNAs in model plants. There remains a notable deficiency in endogenous overexpression or gene knockout studies of miRNAs in bamboo plants themselves. Additionally, there is also a pronounced lack of regulatory network exploration concerning the upstream and downstream components of identified bamboo miRNAs, as well as the precise mechanistic interactions governing these relationships.

Given the substantial species-specific differences in morphological traits and biological characteristics, the mechanisms by which miRNAs regulate organ developmental characteristics are likely to vary across bamboo species. Future studies should broaden the taxonomic scope of bamboo species under investigation to elucidate both the conserved and species-specific aspects of miRNA-mediated regulatory mechanisms. Current efforts in the field are predominantly focused on the identification of miRNA families and their putative target genes, largely through high throughput sequencing and bioinformatic prediction. In subsequent studies, multi-omics data such as transcriptomics, genomics, proteomics, and metabolomics can be integrated to comprehensively dissect the complex networks through which miRNAs regulate bamboo organ development at multiple levels [59,73]. For example, based on multi-omics analysis including RNase R-treated RNA sequencing, small RNA sequencing and customized degradome sequencing, the biogenesis, degradation, and hormone-responsive functions of circRNAs were systematically identified and characterized in moso bamboo [59]. Multi-omics approaches, including genome sequencing, RNA sequencing, and metabolite analysis, were used to investigate bitter flavor formation and transition in the shoot of *Bambusa oldhamii* [73]. Meanwhile, several state-of-the-art approaches, such as single-cell sequencing and spatial transcriptomics, can also be employed to investigate the spatiotemporal expression patterns of miRNAs and their target genes at the cellular level [35,74]. For instance, Qin et al. integrated single-nucleus and spatial transcriptomic atlas to define distinct IcM populations, map their developmental dynamics, and reveal key regulatory networks in moso bamboo [35]. Guo et al. utilized a spatiotemporal transcriptome atlas to elucidate gene regulatory patterns during the organogenesis of rapid growing shoots in moso bamboo [74]. On the other hand, experimental verification of the precise miRNA–target interactions, along with their associated upstream and downstream signaling pathways, also remains highly limited in bamboo organ development to date. Therefore, multiple methods such as RACE (rapid amplification of cDNA ends) and BiFC (bimolecular fluorescence complementation) can be used to validate the mechanistic interaction between miRNAs and TFs. Regarding the lack of endogenous expression of miRNAs in bamboo plants, researchers have successfully developed CRISPR (Clustered Regulatory Interspaced Short Palindromic Repeats)/Cas9-based genome editing system and BaMV (Bamboo Mosaic Virus)-mediated transgene-free genome editing system in moso bamboo and ma bamboo, respectively, which hold great promise for advancing functional genomics and genetic modification in bamboo [75,76,77]. For example, CRISPR-based genome editing system enables the alteration of gene expression levels for key genes governing photosynthesis and lignin production in moso bamboo [75]. BaMV-mediated transformation system allows the efficient delivery of exogenous genes more than 4 kb in length into both moso bamboo and ma bamboo [77]. These advancements facilitate further in-depth studies on miRNA-medicated bamboo organ development, thereby accelerating genetic improvement and innovative breeding of bamboo, as well as the conservation and utilization of bamboo germplasm resources.

## Data Availability

Data sharing is not applicable to this article.

## Abbreviations

The following abbreviations are used in this manuscript:

MiRNAs	MicroRNAs
TF	Transcription factor
SAM	Shoot apical meristem
RAM	Root apical meristem
IcM	Intercalary meristem
CTK	Cytokinin
GA	Gibberellin
SA	Salicylic acid
JA	Jasmonic acid
ABA	Abscisic acid
IAA	Auxin
ID	Initial cell division phase
RD	Rapid cell division phase
RE	Rapid cell elongation phase
DZ	Division zone
EZ	Elongation zone
MZ	Maturation zone
CRISPR	Clustered regulatory interspaced short palindromic repeats
BaMV	Bamboo mosaic virus

## References

[1] Liese W., Köhl M. (2015). Bamboo: The Plant and Its Uses.

[2] Li L., Yu M.H., Yao W.J., Ding Y.L., Lin S.Y. (2023). Research advance in growth and development of bamboo organs. Ind. Crops Prod..

[3] Basak M., Dutta S., Biswas S., Chakraborty S., Sarkar A., Rahaman T., Dey S., Biswas P., Das M. (2021). Genomic insights into growth and development of bamboos: What have we learnt and what more to discover?. Trees.

[4] Zheng A., Pacala S. (2024). Unveiling the mysteries of bamboo: The grass that rises to forest dominance. Bull. Ecol. Soc. Am..

[5] Tripathi A., Yadav S., Nishtha, Nkengnamai M., Thakur A., Singh H. (2024). Bamboo: A fast-growing species to mitigate carbon footprint. Forests and Climate Change.

[6] Zheng X., Lin S.Y., Fu H.J., Wan Y.W., Ding Y.L. (2020). The bamboo flowering cycle sheds light on flowering diversity. Front. Plant Sci..

[7] Zhao X., Yang J., Wang H., Xu H., Zhou Y., Duan L. (2025). MicroRNAs in plants development and stress resistance. Plant Cell Environ..

[8] Samad A.F.A., Sajad M., Nazaruddin N., Fauzi I.A., Murad A.M.A., Zainal Z., Ismail I. (2017). MicroRNA and transcription factor: Key players in plant regulatory network. Front. Plant Sci..

[9] Strader L., Weijers D., Wagner D. (2022). Plant transcription factors—Being in the right place with the right company. Curr. Opin. Plant Biol..

[10] Dong Q., Hu B., Zhang C. (2022). microRNAs and their roles in plant development. Front. Plant Sci..

[11] Guo Z., Yang Y., Yang X. (2025). Fast and furious: The rapid turnover of microRNAs in plants. J. Syst. Evol..

[12] Wang W., Wu Q., Wang N., Ye S., Wang Y., Zhang J., Lin C., Zhu Q. (2025). Advances in bamboo genomics: Growth and development, stress tolerance, and genetic engineering. J. Integr. Plant Biol..

[13] Wang G., Yu F., Wu H., Hu S., Wu S., Pei N., Shi J., Lambers H. (2023). Roots originating from different shoot parts are functionally different in running bamboo, *Phyllostachys glauca*. Funct. Ecol..

[14] Ding Y.L., Lin S.Y., Wei Q., Yao W.J., Que F., Li L. (2022). Research progress in bamboo developmental biology. J. Nanjing For. Univ. (Nat. Sci. Ed.).

[15] Gao G.B., Zhang X.P., Wen X., Zhong H., Pan Y.L., Yang J.L., Wu Z.Z. (2023). Rhizome growth characteristics of different potted *Phyllostachys praecox* ‘Prevernalis’ seedlings. Chin. J. Ecol..

[16] Tong L., Li P.H., Zhou G.M., Zhou Y.F., Li C. (2019). A review of research about rhizome-root system in bamboo forest. J. Zhejiang A&F Univ..

[17] Wang L.L., Zhao Z.Q., Qian J., Sun H.J., Zheng H., Zhou B.Z. (2025). Research on the growth dynamics of new roots in *Phyllostachys edulis* based on minirhizotron technique. J. Bamboo Res..

[18] Zhong Y.B., Jiang X., Lou C. (2014). Research progress in growth rhythm of bamboos. World Bamboo Ratt..

[19] Tong R., Chen Q.B., Zhou B.Z., Tang Y.Q., An Y.F., Ge X.G., Cao Y.H., Yang Z.Y. (2020). Structure and biomechanical properties of underground system of Moso bamboo and Lei bamboo. Acta Ecol. Sin..

[20] Wang L.L., Zhao H.S., Chen D.L., Li L.C., Sun H.Y., Lou Y.F., Gao Z.M. (2016). Characterization and primary functional analysis of a bamboo NAC gene targeted by *miR164b*. Plant Cell Rep..

[21] Xu P., Mohorianu I., Yang L., Zhao H., Gao Z., Dalmay T. (2014). Small RNA profile in moso bamboo root and leaf obtained by high definition adapters. PLoS ONE.

[22] Wang J.W., Wang L.J., Mao Y.B., Cai W.J., Xue H.W., Chen X.Y. (2005). Control of root cap formation by microRNA-targeted auxin response factors in Arabidopsis. Plant Cell.

[23] Geng R.M., Mu C.H., Cheng W.L., Gao J., Cheng Z.H. (2025). Genome-wide identification of the miR156 family in *Phyllostachys edulis* and the potential role of *phe*-*MIR156o* in root, culm and panicle regulation. Ind. Crops Prod..

[24] Xu R., Wang P., Pang Y., Liu H., Zhang T., Li Y., Zhang S. (2025). Involvement of the miR156/SPLs/NLP7 modules in plant lateral root development and nitrogen uptake. Planta.

[25] Jin Q.Y., Peng H.Z., Lin E.P., Li N., Huang D.N., Xu Y.L., Hua X.Q., Wang K.H., Zhu T.J. (2016). Identification and characterization of differentially expressed miRNAs between bamboo shoot and rhizome shoot. J. Plant Biol..

[26] Yuan T., Zhu C., Li G., Liu Y., Yang K., Li Z., Song X., Gao Z. (2022). An integrated regulatory network of mRNAs, microRNAs, and lncRNAs involved in nitrogen metabolism of Moso bamboo. Front. Genet..

[27] Hu T.Y., Kong L.H., Hu S.S., Deng M., Yang G.Y., Wei Q., Yu F. (2023). Emerging insights into the roles of the rhizome-culm system in bamboo shoot development through analysis of non-structural carbohydrate changes. Plants.

[28] Hu S., Dong M., Guo Q. (2025). The transcriptome analysis provides new insights into signaling for bamboo shoot development of sympodial bamboo. Foods.

[29] Wei Q., Jiao C., Guo L., Ding Y., Cao Y., Feng J., Dong X., Mao L., Sun H., Yu F. (2016). Exploring key cellular processes and candidate genes regulating the primary thickening growth of Moso underground shoots. New Phytol..

[30] Yang K., Li L., Lou Y., Zhu C., Li X., Gao Z. (2021). A regulatory network driving shoot lignification in rapidly growing bamboo. Plant Physiol..

[31] Li Z., Xu X., Yang K., Zhu C., Liu Y., Gao Z. (2022). Multifaceted analyses reveal carbohydrate metabolism mainly affecting the quality of postharvest bamboo shoots. Front. Plant Sci..

[32] Wang N., Wang W., Cheng Y., Cai C.Y., Zhu Q. (2023). Uncovering the miRNA-mediated regulatory network involved in Ma bamboo (*Dendrocalamus latiflorus*) de novo shoot organogenesis. Hortic. Res..

[33] Li Y., Zhang D., Zhang S., Lou Y., An X., Jiang Z., Gao Z. (2022). Transcriptome and miRNAome analysis reveals components regulating tissue differentiation of bamboo shoots. Plant Physiol..

[34] Li Y., Vasupalli N., Cai O., Lin X., Wu H. (2023). Network of miR396-mRNA in tissue differentiation in Moso bamboo (*Phyllostachys edulis*). Plants.

[35] Qin N., Liu X., Li S., Sun L., Chen C., Lou W., Wang X., Bao P., Cao B., Zhang H. (2025). Single-nucleus and spatial transcriptomics reveal the cell populations of intercalary meristems in bamboo. Proc. Natl. Acad. Sci. USA.

[36] Wang Y., Sun X., Ding Y., Fei Z., Jiao C., Fan M., Yao B., Xin P., Chu J., Wei Q. (2019). Cellular and molecular characterization of a thick-walled variant reveal a pivotal role of shoot apical meristem in transverse development of bamboo culm. J. Exp. Bot..

[37] Chen M., Guo L., Ramakrishnan M., Fei Z., Vinod K.K., Ding Y., Jiao C., Gao Z., Zha R., Wang C. (2022). Rapid growth of Moso bamboo (*Phyllostachys edulis*): Cellular roadmaps, transcriptome dynamics, and environmental factors. Plant Cell.

[38] Wei Q., Guo L., Jiao C., Fei Z.J., Chen M., Cao J.J., Ding Y.L., Yuan Q.S. (2019). Characterization of the developmental dynamics of the elongation of a bamboo internode during the fast growth stage. Tree Physiol..

[39] Ramakrishnan M., Chen M., Ding Y.L., Wei Q. (2026). Testable four-pillar hypotheses and research priorities for decoding Moso bamboo’s extreme growth. New Phytol..

[40] Lin J., He X., Hu Y., Kuang T., Ceulemans R. (2002). Lignification and lignin heterogeneity for various age classes of bamboo (*Phyllostachys pubescens*) stems. Physiol. Plant..

[41] Wang K.L., Wang B., Hu R., Zhao X., Li H., Zhou G., Song L., Wu A.M. (2019). Characterization of hemicelluloses in *Phyllostachys edulis* (moso bamboo) culm during xylogenesis. Carbohydr. Polym..

[42] He C.Y., Cui K., Zhang J.G., Duan A.G., Zeng Y.F. (2013). Next-generation sequencing-based mRNA and microRNA expression profiling analysis revealed pathways involved in the rapid growth of developing culms in Moso bamboo. BMC Plant Biol..

[43] Wang K.L., Zhang Y., Zhang H., Lin X.C., Xia R., Song L., Wu A.M. (2021). MicroRNAs play important roles in regulating the rapid growth of the *Phyllostachys edulis* culm internode. New Phytol..

[44] Li Y., Zhang S.Q., Zhang D.Q., Li X.P., Gao Z.M., Jiang Z.H. (2022). The *miR166*–mRNA network regulates vascular tissue differentiation in Moso bamboo. Front. Genet..

[45] Zhu C., Lou Y., Yang K., Liu Y., Xiao X., Li Z., Guo D., Sun H., Gao Z. (2022). Integrative analyses of morphology, physiology, and transcriptional expression profiling reveal miRNAs involved in culm color in bamboo. Front. Plant Sci..

[46] Wang S.G. (2017). Bamboo sheath—A modified branch based on the anatomical observations. Sci. Rep..

[47] Jin D., Lyu Z., Wang S., Long H., Zhang C., Wang S.H. (2023). Comparison of anatomical structure of six bamboo species cotyledon organs. J. Nanjing For. Univ. (Nat. Sci. Ed.).

[48] Gao Z., Guo L., Chen M., Yu F., Wei Q. (2021). Characterization of the development dynamics within the linear growth bamboo leaf. Physiol. Plant..

[49] Zhao W., Lv Z., Zhang H., Yue J., Zhang X., Li L., Huang F., Lin S. (2024). Anatomical mechanisms of leaf blade morphogenesis in *Sasaella kogasensis* ‘Aureostriatus’. Plants.

[50] Zhao W.Q., Wang N., Lv Z., Zhang H.J., Lin S.Y. (2026). Coordinated chlorophyll degradation and flavonoid accumulation orchestrate leaf variegation of *Sasaella kogasensis* ‘Aureostriatus’. Plant Cell Environ..

[51] Jiang J., Gao Z., Xiang Y., Guo L., Zhang C., Que F., Yu F., Wei Q. (2022). Characterization of anatomical features, developmental roadmaps, and key genes of bamboo leaf epidermis. Physiol. Plant..

[52] Hu S.S., Kong L.H., Xiao J.H., Hu T.Y., Deng M., Yang G.Y., Yu F. (2024). Study on leaf structure characteristics of *Phyllostachys edulis* and its two variants. J. Bamboo Res..

[53] Wang H., Guo L., Zha R., Gao Z., Yu F., Wei Q. (2022). Histological, metabolomic and transcriptomic analyses reveal mechanisms of cold acclimation of the Moso bamboo (*Phyllostachys edulis*) leaf. Tree Physiol..

[54] Zheng H., Bai Y., Li X., Song H., Cai M., Cheng Z., Mu S., Li J., Gao J. (2022). Photosynthesis, phytohormone signaling and sugar catabolism in the culm sheaths of *Phyllostachys edulis*. Plants.

[55] Chen M., Ju Y., Ahamd Z., Yin Z.F., Ding Y.L., Que F., Yan J.J., Chu J.F., Wei Q. (2021). Multi-analysis of sheath senescence provides new insights into bamboo shoot development at the fast growth stage. Tree Physiol..

[56] Zhao H.S., Wang L.L., Dong L.L., Sun H.Y., Gao Z.M. (2014). Discovery and comparative profiling of microRNAs in representative monopodial bamboo (*Phyllostachys edulis*) and sympodial bamboo (*Dendrocalamus latiflorus*). PLoS ONE.

[57] Wang L.L., Li L.C., Sun H.Y., Zhao H.S., Gao Z.M. (2017). Cloning of *miR164b* precursor from *Phyllostachys edulis* and analysis of its function in leaf morphogenesis. Plant Sci. J..

[58] Zhao H., Chen D., Peng Z., Wang L., Gao Z. (2013). Identification and characterization of microRNAs in the leaf of Ma bamboo (*Dendrocalamus latiflorus*) by deep sequencing. PLoS ONE.

[59] Wang Y., Wang H., Wang H., Zhou R., Wu J., Zhang Z., Jin Y., Li T., Kohnen M.V., Liu X. (2023). Multi-omics of circular RNAs and their responses to hormones in Moso bamboo (*Phyllostachys edulis*). Genom. Proteom. Bioinf..

[60] Fan H., Zhuo R., Wang H., Xu J., Jin K., Huang B., Qiao G. (2022). A comprehensive analysis of the floral transition in ma bamboo (*Dendrocalamus latiflorus*) reveals the roles of DlFTs involved in flowering. Tree Physiol..

[61] Biswas P., Chakraborty S., Dutta S., Pal A., Das M. (2016). Bamboo flowering from the perspective of comparative genomics and transcriptomics. Front. Plant Sci..

[62] Wu C., Cheng Z., Gao J. (2024). Mysterious bamboo flowering phenomenon: A literature review and new perspectives. Sci. Total Environ..

[63] Wang W., Franklin S.B., Lu Z., Rude B.J. (2016). Delayed flowering in bamboo: Evidence from *Fargesia qinlingensis* in the Qinling Mountains of China. Front. Plant Sci..

[64] Sembada A.A., Hanisia R.H., Yuliar Y., Hidayat Y., Sumardi I. (2025). Advances in technology for seed germination of bamboo species. Adv. Bamboo Sci..

[65] Wang X., Liu J., Zhao C., Wang S. (2025). Morphological characteristics of floral organs and their taxonomic significance in 23 species of bamboo from Southwest China. Plants.

[66] Lin S.Y., Wan Y.W., Fu H.J., Zhang L., Jiang M.Y., Yin Z.F., Ding Y.L. (2018). Research on inflorescence establishment and revision of inflorescence type in bamboo plants. J. Nanjing For. Univ. (Nat. Sci. Ed.).

[67] Yao W.J., Li C.Z., Lin S.Y., Wang J.P., Fan T.T., Zhao W.Q. (2023). The structures of floral organs and reproductive characteristics of an ornamental bamboo species, *Pleioblastus pygmaeus*. Hortic. Plant J..

[68] Cheng Z., Hou D., Ge W., Li X., Xie L., Zheng H., Cai M., Liu J., Gao J. (2020). Integrated mRNA, microRNA transcriptome and degradome analyses provide insights into stamen development in Moso bamboo. Plant Cell Physiol..

[69] Zhao X.Y., Wang X.Y., Zhao L., Zhang X.M., Chen S.Y., Ma P.F., Hu X.Y., Li D.Z., Guo Z.H. (2015). Investigating the microRNAomes of two developmental phases of *Dendrocalamus latiflorus* (Poaceae: Bambusoideae) inflorescences. Plant Mol. Biol. Rep..

[70] Gao J., Ge W., Zhang Y., Cheng Z., Li L., Hou D., Hou C. (2015). Identification and characterization of microRNAs at different flowering developmental stages in moso bamboo (*Phyllostachys edulis*) by high-throughput sequencing. Mol. Genet. Genom..

[71] Ge W., Zhang Y., Cheng Z., Hou D., Li X., Gao J. (2017). Main regulatory pathways, key genes and microRNAs involved in flower formation and development of moso bamboo (*Phyllostachys edulis*). Plant Biotechnol. J..

[72] Yao W., Shen P., Yang M., Meng Q., Zhou R., Li L., Lin S. (2024). Integrated analysis of microRNAs and transcription factor targets in floral transition of *Pleioblastus pygmaeus*. Plants.

[73] Jiao Y.L., Hu N., Xia H.T., Li X.W., Li J., Liu Y., Wang J.W. (2025). Multi-omics approaches investigate the bitter flavor in the shoot of *Bambusa oldhamii* Munro. BMC Plant Biol..

[74] Guo J., Luo D., Chen Y., Li F., Gong J., Yu F., Zhang W., Qi J., Guo C.C. (2024). Spatiotemporal transcriptome atlas reveals gene regulatory patterns during the organogenesis of the rapid growing bamboo shoots. New Phytol..

[75] Huang B., Zhuo R., Fan H., Wang Y., Xu J., Jin K., Qiao G. (2022). An efficient genetic transformation and CRISPR/Cas9-based genome editing system for Moso bamboo (*Phyllostachys edulis*). Front. Plant Sci..

[76] Wu L., Yang J., Gu Y., Wang Q., Zhang Z., Guo H., Zhao L., Zhang H., Gu L. (2025). Bamboo mosaic virus-mediated transgene-free genome editing in bamboo. New Phytol..

[77] Jin Y., Wang B., Bao M., Li Y., Xiao S., Wang Y., Zhang J., Zhao L., Zhang H., Hsu Y.H. (2023). Development of an efficient expression system with large cargo capacity for interrogation of gene function in bamboo based on bamboo mosaic virus. J. Integr. Plant Biol..

